# Corrigendum to: SUPPLEMENT, Two Decades of Public Health Achievements in Lymphatic Filariasis (2000–2020): Reflections, Progress and Future Challenges

**DOI:** 10.1093/inthealth/ihab014

**Published:** 2021-03-22

 In the originally published version of this supplement, errors relating to the incorrect use of the Merck and MSD corporate names were noted and are listed in this corrigendum. This corrigendum addresses the changes that have subsequently been made to the following articles:

**Setting the stage for a Global Programme to Eliminate Lymphatic Filariasis: the first 125 years (1875–2000)**.

**Eric A Ottesen, John Horton, International Health, Volume 13, Issue Supplement_1, January 2021, Pages S3–S9, https://doi.org/10.1093/inthealth/ihaa061**.

In the Abstract, mention of ‘Merck’ should read: ‘MSD (trade name of Merck & Co. Inc., Kenilworth, NJ, USA)’.

All mention of ‘Merck’ should read: ‘MSD’ in the following areas: under the ‘Ivermectin and albendazole’ heading; in the ‘Resources’ section; in ‘The launch’ section; and in the Acknowledgements.

**The role of medicine donations in the global programme for the elimination of lymphatic filariasis**.

**Tijana Williams, Rachel Taylor, Minne Iwamoto, Takayuki Hida, Fabian Gusovsky, International Health, Volume 13, Issue Supplement_1, January 2021, Pages S39–S43, https://doi.org/10.1093/inthealth/ihaa077**.

In the Abstract, mention of ‘MSD (trade name of Merck & Co., Kenilworth, NJ, USA)’ should read: ‘MSD (trade name of Merck & Co. Inc., Kenilworth, NJ, USA’.

**A triple-drug treatment regimen to accelerate elimination of lymphatic filariasis: From conception to delivery**.

**Gary J Weil, Julie A Jacobson, Jonathan D King, International Health, Volume 13, Issue Supplement_1, January 2021, Pages S60–S64, https://doi.org/10.1093/inthealth/ihaa046**.

The section heading ‘Policy changes at WHO and at Merck Sharp & Dohme (MSD)’ should read: ‘Policy changes at WHO and at MSD’. In addition, mention of ‘MSD’ in the ‘Policy changes at WHO and at MSD’ section should read: ‘MSD (trade name of Merck & Co. Inc., Kenilworth, NJ, USA)’.

**Evolution of the monitoring and evaluation strategies to support the World Health Organization's Global Programme to Eliminate Lymphatic Filariasis**.

**Patrick J Lammie, Katherine M Gass, Jonathan King, Michael S Deming, David G Addiss, Gautam Biswas, Eric A Ottesen, Ralph Henderson, International Health, Volume 13, Issue Supplement_1, January 2021, Pages S65–S70, https://doi.org/10.1093/inthealth/ihaa084**.

In the ‘Scaling Up’ section, mention of ‘Merck’ should read: ‘MSD (trade name of Merck & Co. Inc., Kenilworth, NJ, USA)’.

